# Whole-Genome Analysis Deciphers Population Structure and Genetic Introgression Among Bovine Species

**DOI:** 10.3389/fgene.2022.847492

**Published:** 2022-05-27

**Authors:** Rong Li, Shanyuan Chen, Chunqing Li, Heng Xiao, Vânia Costa, Mohammad Shamsul Alam Bhuiyan, Mumtaz Baig, Albano Beja-Pereira

**Affiliations:** ^1^ School of Ecology and Environmental Science, Yunnan University, Kunming, China; ^2^ College of Life Science, Yunnan Normal University, Kunming, China; ^3^ Centro de Investigação em Biodiversidade e Recursos Genéticos (CIBIO-InBIO), Universidade do Porto, Vairão, Portugal; ^4^ Department of Animal Breeding and Genetics, Bangladesh Agricultural University, Mymensingh, Bangladesh; ^5^ Department of Zoology, Government Vidarbha Institute of Science and Humanities, Amravati, India; ^6^ Ambiente e Ordenamento do Território (DGAOT), Faculdade de Ciências, Universidade do Porto, Porto, Portugal; ^7^ Sustainable Agrifood Production Research Centre (GreenUPorto), University of Porto, Vairão, Portugal

**Keywords:** *Bos* species, population structure, genetic introgression, selection signature, high-altitude adaptation

## Abstract

It is known that throughout history and presently, taurine (*Bos taurus*) and indicine/zebu (*Bos indicus*) cattle were crossed with other bovine species (e.g., gayal, gaur, banteng, yak, wisent, and bison). Information on the role of interspecific hybridization to facilitate faster adaptation of the newly arrived domestic species to new environments is poorly known. Herein, we collected 266 samples of bovine species of the taurine, zebu, yak, and gaur from West Europe, Southwest China, Indian subcontinent, and Southeast Asia to conduct the principal component analysis (PCA), admixture, gene flow, and selection signature analyses by using SNPs distributed across the bovine autosomes. The results showed that the genetic relationships between the zebu, yak, and gaur mirrored their geographical origins. Three ancestral components of the European taurine, East Asian taurine, and Indian zebu were found in domestic cattle, and the bidirectional genetic introgression between the Diqing cattle and Zhongdian yak was also detected. Simultaneously, the introgressed genes from the Zhongdian yak to the Diqing cattle were mainly enriched with immune-related pathways, and the *ENPEP*, *FLT1*, and *PIK3CA* genes related to the adaptation of high-altitude hypoxia were detected. Additionally, we found the genetic components of the Zhongdian yak had introgressed into Tibetan cattle. The 30 selected genes were detected in Tibetan cattle, which were significantly enriched in the chemokine signaling pathway. Interestingly, some genes (*CDC42*, *SLC39A2*, and *EPAS1*) associated with hypoxia response were discovered, in which *CDC42* and *SLC39A2* played important roles in angiogenesis and erythropoiesis, and heart function, respectively. This result showed that genetic introgression was one of the important ways for the environmental adaptation of domestic cattle.

## Introduction

The genus *Bos* belongs to the subfamily Bovidae and includes several species, such as *Bos taurus* (taurine), *Bos indicus* (indicine/zebu), *Bos frontalis* (gayal), *Bos gaurus* (gaur), *Bos javanicus* (banteng), and *Bos grunniens* (yak) (Wu et al., 2018), which play significant roles in human history. The high-throughput genome sequencing effort has revealed new and helpful insights on the domestication and environmental adaptation mechanisms operating on these *Bos* species (Hu et al., 2012; Qiu et al., 2012; Gautier et al., 2016; Chen et al., 2018; Wu et al., 2018).

Information from high-density genome-wide SNP panels has been used to classify the ancestral components and reconstruct the historical migration routes of domestic cattle in the world (Decker et al., 2014). For example, a previous study found that all Eurasian cattle stem from five ancestral components: European taurine, Eurasian taurine, East Asian taurine, Chinese indicine, and Indian indicine (Chen et al., 2018). Yet, little is known about the contribution of other *Bos* species into this genome architecture.

The domestication of *B. taurus* and *B. indicus* occurred in the Near East and Indus Valley, respectively. There is no reproductive isolation between the taurine and zebu, the two types of cattle can be hybridized and their offspring are completely fertile (Wei et al., 2016). Studies showed that domestic cattle crossed with other *Bos* species broke reproductive isolation by hybridization or backcrossing, resulting in genetic introgression among *Bos* species (Chen et al., 2018; Wu et al., 2018). It is expected that introgression will help *Bos* species to adapt faster to the local environment. Gao et al. used a model-based clustering and *f4*-statistics analyses to quantify banteng and gayal introgression into southern Chinese cattle (Gao et al., 2017). Bidirectional gene introgression between the yak and cattle was also detected from whole-genome sequencing analysis (Wu et al., 2018). Finally, the latter study also found signs of introgression between the yak and Tibetan cattle at genes related with hypoxia adaptation (*EGLN1*, *EGLN2*, and *HIF3a*) and other study and quantified the proportion of taurine cattle introgression (∼1.3%) in the yak genome (Medugorac et al., 2017). As demonstrated in other studies, high-density BovineHD BeadChip genotyping arrays can be useful tools to estimate for population structure, genetic introgression, and selection signatures of *Bos* species (Medugorac et al., 2017; Mukherjee et al., 2018). In this study, we analyzed a total of 266 individuals of 10 populations of four *Bos* species (taurine, zebu, yak, and gaur) from West Europe, Southwest China, Indian subcontinent, and Southeast Asia by using the Illumina BovineHD BeadChip. The main purposes of this study were to 1) examine whether the genetic relationships of these *Bos* species reflect their geographical origins; 2) detect genetic admixture and genetic introgression among these cattle populations; and 3) screen out the candidate genes related to high-altitude adaptation of Tibetan cattle.

## Materials and Methods

### Ethics Approval Statement

Ear skin tissue samples were collected by veterinary practitioners with the permission and in presence of the owners. Veterinarians followed standard procedures and relevant national guidelines to ensure appropriate animal care. This study was approved by the Committee on Animal Research and Ethics of Yunnan University (ynucae20190011).

### Sample Collection and Genomic DNA Extraction

We collected a total of 266 samples of 10 populations of four *Bos* species (Table 1), including Holstein (*n* = 34), Diqing cattle (*n* = 16), Tibetan cattle (*n* = 39), Indian zebu (*n* = 25), Bangladesh zebu (*n* = 21), Myanmar cattle (*n* = 38), Zhongdian yak (*n* = 13), Tibetan yak (*n* = 58), Maiwa yak (*n* = 8), and Gaur (*n* = 14). Diqing cattle and Zhongdian yak live in Shangri-La, Diqing Prefecture, which is at an average altitude of 3,300 m. All samples were divided into four geographic regions: West Europe, Southwest China, Indian subcontinent, and Southeast Asia. Genomic DNA was extracted from ear skin tissues using the DNeasy Blood & Tissue kit (Qiagen).

**TABLE 1:** Sample information of 266 samples from 10 bovine populations.

Species	Population (abbreviation)	Number of samples	Origin regions	Geographical region
*Bos taurus*	Holstein (HOL)	34	Netherlands	West Europe
	Diqing cattle (DQ)	16	Yunnan Province, China	Southwest China
	Tibetan cattle (TIC)	39	Tibet, China	Southwest China
*Bos indicus*	Indian zebu (IND)	25	India	Indian subcontinent
	Bangladesh zebu (BAN)	21	Bangladesh	Indian subcontinent
	Myanmar cattle (MYA)	38	Myanmar	Southeast Asia
*Bos grunniens*	Zhongdian yak (ZDY)	13	Yunnan Province, China	Southwest China
	Tibetan yak (TIY)	58	Tibet, China	Southwest China
	Maiwa yak (MWY)	8	Sichuan Province, China	Southwest China
*Bos gaurus*	Gaur (GAU)	14	India	Indian subcontinent
	Total	266		

### Quality Control Procedures

Genotyping was carried out using the Illumina BovineHD BeadChip (777,962 SNPs) (Rincon et al., 2011). Markers were filtered to exclude the unmapped UMD3.1 bovine genome assembly (Zimin et al., 2009), X, Y, and mitochondrial chromosomes. Finally, 735,293 autosomal SNPs were used for quality control analysis. During quality control, markers with a call rate less than 90% and minor allele frequency (MAF) lower than 0.05 and significantly deviated from Hardy Weinberg equilibrium (*p* < 0.001) were excluded for all analysis based on previous research (Uzzaman et al., 2014). Finally, 638,034 SNPs were excluded, leaving 97,259 SNPs for downstream analyses.

### Genomic Diversity Analysis

The observed heterozygosity (*Ho*), expected heterozygosity (*He*), and inbreeding coefficient (*f*) for each population of four *Bos* species were calculated using PLINK v1.90 (Purcell et al., 2007; Chang et al., 2015). Moreover, heterozygosity estimates are sensitive to various ascertainment biases when SNPs discovered in one breed are used to genotype other breeds (Nielsen, 2004; Edea et al., 2015). Analyses of diversity indexes based on ascertained SNP data would produce false positive results (Lachance and Tishkoff, 2013). Previous studies demonstrated that pruning of SNPs in high linkage disequilibrium (LD) would reduce the influence of ascertainment biases (López Herráez et al., 2009; Kijas et al., 2012). Therefore, we used SNP data sets after quality control and LD-pruning to calculate the genomic diversity indexes.

### Principal Component and Admixture Analyses

Before performing the principal component analysis (PCA) and admixture analyses, the autosomal SNPs data were further pruned for linkage disequilibrium (LD) higher than 0.5 using a sliding window approach of 50 SNPs and a step size of 5 SNPs (50 5 0.5) in PLINK v1.9 (Purcell et al., 2007; Chang et al., 2015). The PCA was executed using the SMARTPCA program in EIGENSOFT v6.1.4 (Price et al., 2006) to discern genetic relationships among three data sets: 1) all 266 samples from 10 populations; 2) all 79 yak samples, including 58 Tibetan yak, 13 Zhongdian yak, and 8 Maiwa yak from Southwest China; 3) all 84 zebu samples, including 38 Myanmar cattle from Southeast Asia and 25 Indian zebu and 21 Bangladesh zebu from the Indian subcontinent. Furthermore, an identity-by-state (IBS) matrix of genetic distance containing each pairwise combination of all individuals was generated by using PLINK v1.9 referred to a previously study (Zhou et al., 2016). We constructed a neighbor-joining (NJ) tree based on the IBS matrix in MEGA v7.0 (Kumar et al., 2016).

To further determine the relative contribution of different potential ancestors on the genomic structure of these 10 bovine populations, population admixture analysis was carried out by using ADMIXTURE v1.30 (Alexander et al., 2009). Additionally, the corresponding cross-validation error value of cluster (K = 1–10) was calculated in ADMIXTURE v1.30. These results of PCA and admixture analyses were visualized using the R package ggplot2 (Wickham, 2016).

### Gene Flow Detection

To detect gene flow among populations, we used the three-population test (*f3* statistics) and calculated their corresponding normalized value (z-scores) in the “qp3Pop” program implemented in the ADMIXTOOLS v6.0 (Patterson et al., 2012). The *f3* statistics considers triplet of the populations (A, B, and C), where C is the test (target) population with A and B as reference (source) populations. If the z-score (z ≤−3.0) is significantly negative, showing the test population C has admixture from both the reference populations A and B.

To assess the direction of gene flow, the *D* statistics were implemented in the ADMIXTOOLS v6.0. The *D* statistics method considers the tree topology [[[W, X], Y], Z], where Z represents outgroup. The gaur population (14 individuals) was selected as outgroup based on previous similar research (Barbato et al., 2020). Y is the admixture population, and W and X are the test populations. The *D* statistics method counts the “ABBA” sites, where W and Z share the outgroup allele (A) and X and Y share the derived allele (B) as well as the “BABA” sites, where W and Y shares the derived allele and X and Z shares the outgroup allele. Admixture between Y and either of the test populations creates a significant difference between the ABBA and BABA counts, with a z-score >3.0 (gene flow between W and Y) or ≤ −3.0 (gene flow between X and Y).

To observe migration events among 10 populations, we constructed a maximum likelihood (ML) tree by using the TREEMIX v1.12 (Pickrell et al., 2012). We set gaur as the root and grouped together 1,000 SNPs to account for the linkage disequilibrium and used 5,000 SNPs to calculate standard errors of migration proportions based on a previous study (Wu et al., 2018). The migration edges were added to the ML tree until 99.8% of the variance of the ancestry between the populations was explained. After building the ML tree, we respectively allowed 1–6 migration events in the tree to draw the ML graphs and corresponding residuals.

### Genetic Introgression Detection and Enrichment Analyses

To further detect the genomic regions of potential introgression among these 10 populations of *Bos* species, the genomic local ancestry was inferred using PCAdmix v1.0 (Brisbin et al., 2012). This software scans the target genome using a sliding window approach to estimate the relative ancestry proportions of the reference populations for each chromosome and haploid individual. Before running the PCAdmix software, genotypes of target population and reference population were phased using default parameters in BEAGLE v4.1 (Browning and Browning, 2013). Due to the absence of genetic distance, we assumed that the genetic distance was equal to physical distance (1 Mb ≈ 1 cM) (Rothammer et al., 2013). Gene annotation was performed for the introgression regions among populations based on the UMD3.1 reference genome. Additionally, to understand biological functions of introgressed genes, the Gene Ontology (GO) and Kyoto Encyclopedia of Genes and Genomes (KEGG) functional enrichment analyses were executed using the DAVID v6.8 (https://david-d.ncifcrf.gov/). Among the GO enrichment analysis included Molecular function (MF), Cellular component (CC) and Biological process (BP).

### Selection Signature Analysis

To detect genes under positive selection in Tibetan cattle, Holstein as a reference population of a lowland taurine breed was used to investigate selection signatures in Tibetan cattle by using the population differentiation index (*F*_ST_), cross-population extended haplotype homozygosity (XP-EHH), and cross-population composite likelihood ratio (XP-CLR) methods. The VCFTOOLS v0.1.15 (Danecek et al., 2011) was used to estimate the *F*_ST_ values using non-overlapping 50 kb windows sliding in 10 kb increments, and the result showed that the negative and missing *F*_ST_ values were discarded because these values have no biological interpretation (Akey et al., 2002).

Before running the XP-EHH and XP-CLR methods, the haplotypes were reconstructed for every chromosome using default parameters in BEAGLE v4.1 (Browning and Browning, 2013). The XP-EHH values was computed in SELSCAN v1.1.0 (Szpiech and Hernandez, 2014), then all XP-EHH values were normalized using the norm program implemented in SELSCAN v1.1.0b. Finally, the standardized XP-EHH values were sorted from largest to smallest; the SNP corresponding to the top 1% XP-EHH values was selected as the core site, and the upstream and downstream extensions were each extended by 50 kb as the selected regions. For the XP-CLR scores were computed in XP-CLR v1.0 (Chen H. et al., 2010). We used the non-overlapping sliding windows of 50 kb and a maximum number of 600 SNPs within each window to calculate the XP-CLR values. Furthermore, we downweighted the correlation level from which the SNPs contribute to XP-CLR results to 0.95. The regions with XP-CLR values in the top 1% of the empirical distribution were considered as candidate signatures, and the genes that span the window regions were defined as candidate genes (Taye et al., 2018).

All markers with top 1% *F*_ST_, XP-EHH, and XP-CLR values were considered to represent a selection signature, and then gene annotation was performed based on the UMD3.1. We carried out the functional enrichment analysis and detected important candidate genes related to high-altitude adaptation by literature review.

## Results

### Genomic Diversity Index

We used both the 97,259 SNPs and 64,704 SNP data sets to calculate the genomic diversity index of 10 bovine populations (Table 2). For 97,259 SNP data sets, the results showed that the average *Ho* ranged from 0.029 in the Tibetan yak to 0.396 in Holstein and the average *He* ranged from 0.028 in the Tibetan yak/Maiwa yak to 0.362 in Holstein. Similarly, using the 64,704 SNP data sets, the lowest and highest heterozygosities were found in the Tibetan yak and Holstein, respectively. Furthermore, the minimum and maximum levels of inbreeding were found in Holstein and gaur population, respectively.

**TABLE 2:** Genetic diversity index among *Bos* species.

Species	Population	Number	97,259 SNPs			64,704 SNPs		
			*Ho*	*He*	*f*	*Ho*	*He*	*f*
*Bos taurus*	Holstein	34	0.396	0.362	−0.091	0.392	0.357	−0.093
	Diqing cattle	16	0.331	0.312	−0.059	0.334	0.316	−0.056
	Tibetan cattle	39	0.348	0.335	−0.039	0.351	0.338	−0.038
*Bos indicus*	Indian zebu	25	0.231	0.220	−0.048	0.254	0.242	−0.050
	Bangladesh zebu	21	0.227	0.214	−0.056	0.249	0.235	−0.058
	Myanmar cattle	38	0.226	0.216	−0.048	0.249	0.238	−0.051
*Bos grunniens*	Zhongdian yak	13	0.045	0.043	−0.050	0.053	0.050	−0.055
	Tibetan yak	58	0.029	0.028	−0.034	0.037	0.036	−0.040
	Maiwa yak	8	0.031	0.028	−0.079	0.039	0.036	−0.092
*Bos gaurus*	Gaur	14	0.085	0.085	−0.012	0.099	0.098	−0.014

*Ho*, observed heterozygosity; *He*, expected heterozygosity; *f*, inbreeding coefficient.

### Population Structure of *Bos* Species

**FIGURE 1:**
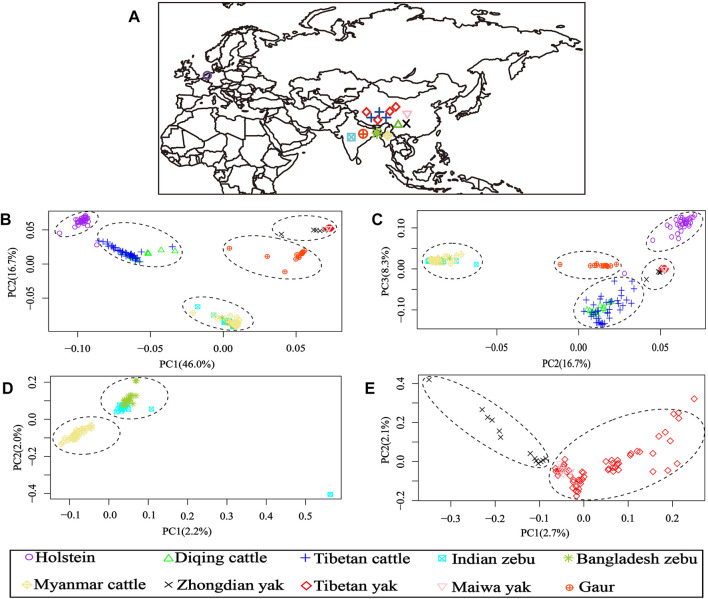
Geographical distribution and principal component analysis (PCA) results of 10 bovine populations. **(A)** Region information of 10 populations; **(B)** PCA results of PC1 vs. PC2; **(C)** PCA results of PC2 vs. PC3; **(D)** PCA results of 84 samples from all zebu populations (Indian zebu, Bangladesh zebu, and Myanmar cattle); **(E)** PCA results of 79 samples from all yak populations (Tibetan yak, Zhongdian yak, and Maiwa yak).

After LD-pruning, the 64,704 SNP data sets were used for the PCA and admixture analyses. The PCA results of 10 cattle populations from four *Bos* species are shown in Figure 1. The first (PC1) and second (PC2) principal components accounted for 46.0% and 16.7% of the total variation, respectively. The PCA clearly separated the individuals into five clusters: one group consisting of Holstein cattle, second cluster formed by Diqing cattle and Tibetan cattle, third including all zebu cattle, fourth gathering all yak populations, and fifth groups constituted by gaur individuals (Figure 1B). Furthermore, the PC2 and third (PC3) principal components accounted for 16.7% and 8.3% of the total variation, which was similar to the results of PC1 and PC2 (Figure 1C).

**FIGURE 2:**
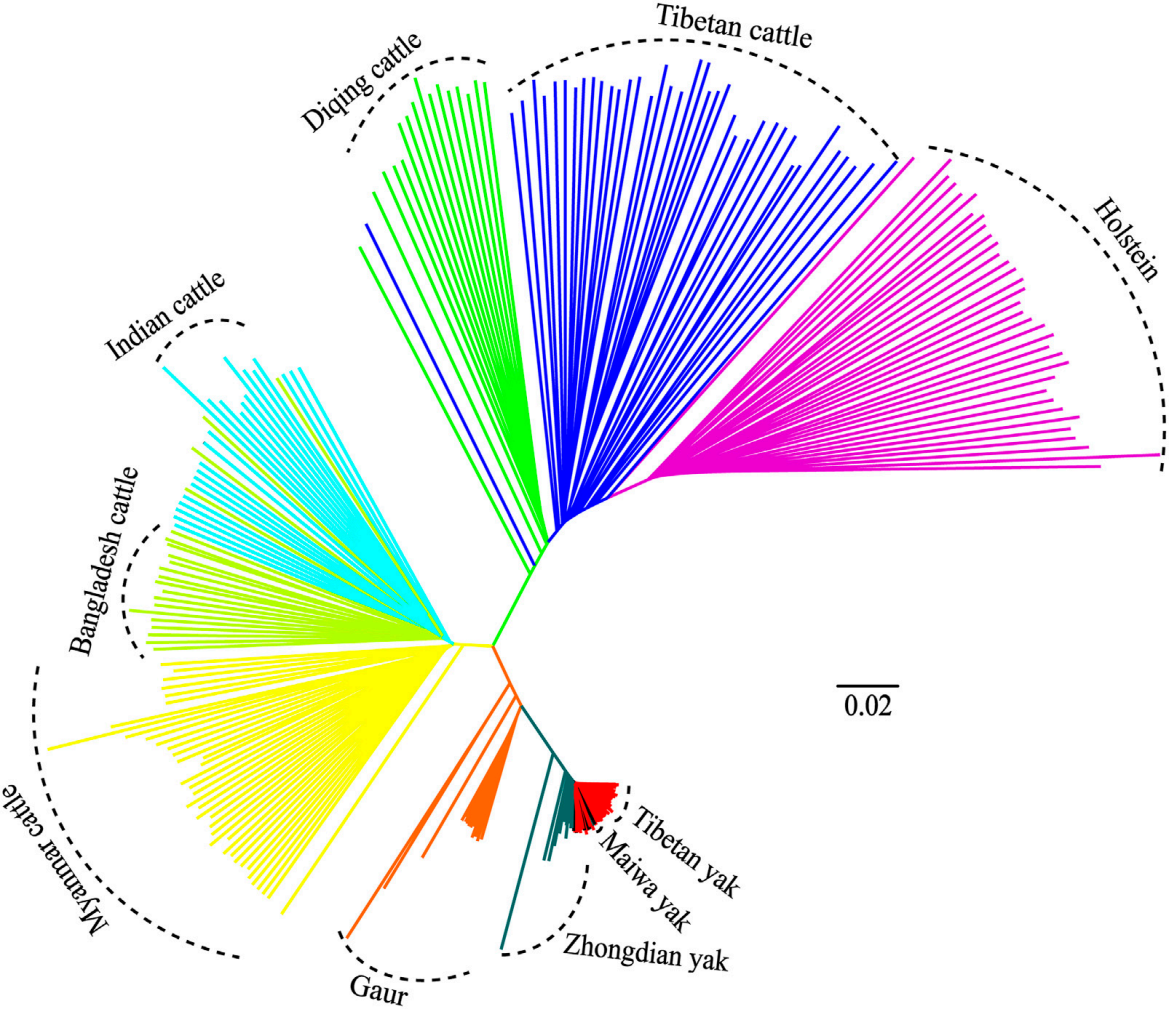
Neighbor-joining (NJ) tree of 10 populations.

Additionally, the genetic relationships between the zebu, yak, and gaur mirrored their geographical origins (Figure 1A). For zebu population, the PC1 accounted for 2.2% and PC2 explained 2.0% of the total variation. The PCA divided the zebu population into a distinct group formed by Myanmar individuals and a combined cluster including Indian and Bangladesh samples (Figure 1D). For the yak population, we found that PC1 and PC2 accounted for 2.7% and 2.1% of the total variation, respectively. The PC1 clearly separated the Zhongdian yak from the Tibetan yak and Maiwa yak (Figure 1E). These PCA results (Figures 1B,C) were strongly supported by the population clustering observed in a neighbor-joining tree (Figure 2).

**FIGURE 3:**
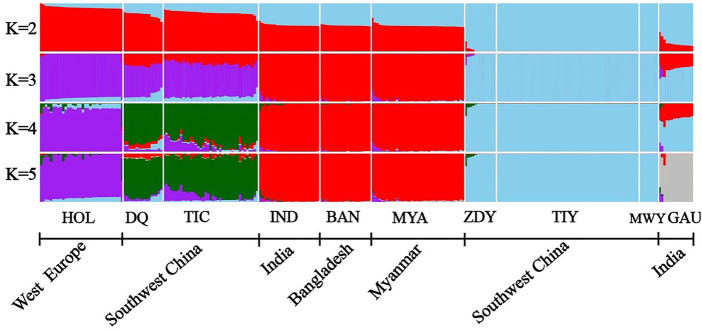
ADMIXTURE analyses of 10 populations (*K* = 2–5). The bovine population abbreviations are given in Table 1.

We performed the admixture analysis among populations based on 64,704 SNPs and obtained patterns of admixture for each population in our study (Figure 3). When K = 2, the yak population (“sky blue”) from southwestern China were first separated from other populations. Subsequently, the zebu population (“red”) was separated from other populations at K = 3. When K = 4, the Holstein population (“purple”) were separated from Diqing cattle and Tibetan cattle (“green”). The lowest cross-validation error value (0.35968) was detected at K = 5 (Supplementary Figure S1), all individuals formed five clusters, including Holstein as a cluster, Diqing cattle and Tibetan cattle from Southwest China as another cluster, the remaining zebu population, yak population, and gaur population (“grey”) each clustered together. These results were consistent with those of the PCAs (Figures 1B,C). Interestingly, we observed genetic components of the yak population may have introgressed into Diqing cattle and Tibetan cattle at K = 4–5, while the Zhongdian yak has genetic components of Diqing cattle/Tibetan cattle. Additionally, we found that three ancestral components of the European taurine (e.g., Holstein), East Asian taurine (e.g., Diqing cattle and Tibetan cattle), Indian-type zebu (Indian zebu, Bangladesh zebu, and Myanmar cattle) in domestic cattle by combining the PCA and NJ tree results. It should be noted that Indian zebu, Bangladesh zebu, and Myanmar cattle from different geographical regions formed a distinct cluster (Figure 3).

### Diqing Cattle and Zhongdian Yak Have a Relevant Gene Flow

In order to further test the gene flow between Diqing cattle and yak. The *f3* statistics analysis was performed by using Diqing cattle as a target and Holstein (or Tibetan cattle) and other Bovine species as the source population. When using the Zhongdian yak and Holstein as the sources has a significant z-score (−5.754), showing that there is gene flow between Diqing cattle and Zhongdian yak (Supplementary Table S1). Additionally, the *D* statistics was performed following the tree topology [[[Holstein, Diqing cattle], Zhongdian yak], Gaur] and found a significant z-score (−13.164) (Supplementary Table S2), displaying that Zhongdian yak introgression into Diqing cattle. TreeMix analysis (*m* = 5) also confirmed the gene flow from the Zhongdian yak to Diqing cattle (Supplementary Figure S2).

The same methods (*f3* statistics, *D* statistics, and TreeMix analyses) were used to detect the genetic introgression from Diqing cattle to the Zhongdian yak. When using the Tibetan yak (or Maiwa yak) and Diqing cattle as the source populations, it had a significant z-score (−17.987/−14.108) for *f3* statistics (Supplementary Table S3). Furthermore, the *D* statistics was executed following the tree topology [[[Tibetan yak, Zhongdian yak], Diqing cattle], Gaur] and had a significant z-score (−15.981) (Supplementary Table S4), showing that Diqing cattle possibly had introgressed into the Zhongdian yak. The TreeMix results (*m* = 4) also confirmed the gene flow from the Diqing cattle to the Zhongdian yak (Supplementary Figure S2). The bidirectional genetic introgression between the Diqing cattle and Zhongdian yak was detected based on aforementioned research results.

### Diqing Cattle Has the Genetic Components of Zhongdian Yak

We selected Diqing cattle as the target population, and the Zhongdian yak and Holstein as the reference populations to detect the introgressed proportion from the Zhongdian yak into Diqing cattle using PCAdmix v1.0. We found that the average introgressed proportion was 2.37% (0.84%–7.68%) from the Zhongdian yak into Diqing cattle (Supplementary Table S5). The average introgressed proportion was 5.04% (0.83%–21.25%) from Diqing cattle into the Zhongdian yak (Supplementary Table S6). To identify the biological function of the introgression regions from the Zhongdian yak into Diqing cattle, we exploited the gene of 402 introgressed fragments that were shared by at least two haplotypes in Diqing cattle. The introgressed genes were enriched for GO terms and KEGG pathways (Supplementary Table S7, *p* < 0.01), the results displayed that the GO terms mainly include CCR chemokine receptor binding (GO:0048020), chemokine activity (GO:0008009), inflammatory response (GO:0006954), and lymphocyte chemotaxis (GO:0048247). The KEGG pathways were mainly involved in the chemokine signaling pathway (bta04062) and leukocyte transendothelial migration (bta04670), and these terms and pathways are associated with immunity. Furthermore, to test whether the genetic introgression of the Zhongdian yak influences Diqing cattle to high-altitude and hypoxia adaptations, we found that some key genes related to high-altitude and hypoxia adaptations, such as *ENPEP* (Eichstaedt et al., 2015; Wang et al., 2016; Wei et al., 2016), *FLT1* (Kraft et al., 2016), and *PIK3CA* (Shimomura et al., 2013).

### Tibetan Cattle Have the Genetic Components of Zhongdian Yak

Tibetan cattle mainly live in Tibetan areas with an altitude of 2,300–3,800 m. Studies have shown that Tibetan cattle have obtained gene alleles from the yak through genetic introgression (Chen et al., 2018; Wu et al., 2018). To further detect whether there was genetic introgression from the yak into Tibetan cattle, we performed the *f*3 statistics, *D* statistics, and TreeMix analyses. When using the Zhongdian yak and Holstein as the source populations has a significant z-score (−6.877) for *f*3 statistics, it shows that there is gene flow between Tibetan cattle and Zhongdian yak (Supplementary Table S8). The *D* statistics was performed following the tree topology [[[Holstein, Tibetan cattle], Zhongdian yak], Gaur] and found a significant z-score (−10.250) (Supplementary Table S9), displaying that Zhongdian yak had introgressed into Tibetan cattle. Furthermore, the TreeMix analysis (*m* = 5) also confirmed the gene flow from the Zhongdian yak to Tibetan cattle (Supplementary Figure S2).

**FIGURE 4:**
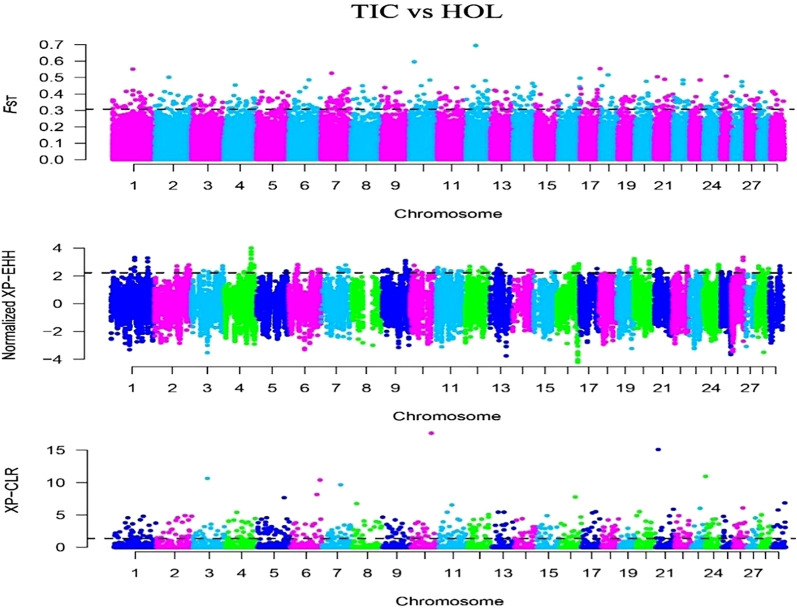
Genome-wide distribution of *F*_ST_, XP-EHH, and XP-CLR of Tibetan cattle vs. Holstein on autosomes. The horizontal black line in the graph indicates the values of the top 1%.

In this study, we selected the Tibetan cattle (39 individuals) as target population and Holstein (34 individuals) of lowland as reference population. The *F*_ST_, XP-EHH, and XP-CLR methods were used to detect selection signatures on the Tibetan cattle genome. Manhattan plots of the genome-wide *F*_ST_, XP-EHH, and XP-CLR analyses are depicted in Figure 4. Finally, 270, 162, and 296 genes were identified from the *F*_ST_, XP-EHH, and XP-CLR methods, respectively. The 30 common genes were obtained in at least two methods, of which *CDC42* and *SLC39A2* genes were found to be associated with high-altitude adaptation. The *EPAS1* related to high-altitude adaptation was detected in the separate XP-EHH method. Additionally, we executed the GO and KEGG analyses for the 30 common genes (Table 3) and found that the GO terms were mainly enriched in chemotaxis (GO:0006935) and small GTPase-mediated signal transduction (GO:0007264). The KEGG pathway mainly enriched in chemokine signaling pathway (bta04062) (*p* < 0.05).

**TABLE 3:** Enrichment pathways of the 30 common genes in Tibetan cattle vs. Holstein.

Category	Term	Count	p-value
BP	GO:0006935∼chemotaxis	3	0.0016
BP	GO:0007264∼small GTPase-mediated signal transduction	3	0.0243
KEGG	bta04062:Chemokine signaling pathway	3	0.0192

## Discussion

In this study, a total of 266 individuals of 10 populations of four *Bos* species were used to explore their population structure and genetic introgression. The PCA showed that all individuals formed five clusters (Figures 1B,C), and the genetic relationship among other three *Bos* species mirrored their geographic origins except taurine based on the 64,704 SNP data sets (Figure 1A). Furthermore, the PCA and a neighbor-joining (NJ) tree showed that the Indian zebu and Bangladesh zebu and Tibetan yak and Maiwa yak were clustered into a mixed cluster, respectively. It is possible that their geographical connection leads to frequent gene flow. The admixture analysis displayed that all samples formed five clusters, which were consistent with the PCA results, illustrating the reliability of clustering results. We observed European taurine, East Asian taurine, and Indian zebu ancestral components in domestic cattle by performing the admixture analysis; the result was similar to a previous study based on the whole-genome re-sequencing analysis (Chen et al., 2018), and it further showed that the genome-wide SNP chip is reliable for the classification of ancestral components. Studies have shown that zebu may have dispersed from the Indian subcontinent to East Asia about 3,500–2,500 years before present (YBP) (Chen S. et al., 2010), passed Southeast Asia (e.g., Myanmar), and entered into southwestern China (Higham, 1996). Therefore, it can be used to explain that Indian zebu, Bangladesh zebu, and Myanmar cattle from different geographical regions gathered in a separate cluster.

*Bos* species can provide production and living resources for human beings, including meat, milk, leather, and fuel. Thus, human migration also drove the continuous migration of different *Bos* species in history, leading to frequent gene flow among these bovine populations. For example, genetic introgression from the yak into Tibetan cattle (Chen et al., 2018; Wu et al., 2018), Bali cattle introgressed into Chinese Hainan and Luxi cattle breeds (Decker et al., 2014), and domestic cattle (taurine and zebu) introgressed into the yak (Medugorac et al., 2017). These studies demonstrated that genetic introgression can help domestic cattle better adapt to extreme environments (e.g., tropical climate and high-altitude environments). Previous studies have shown that genetic component of the yak introgressed into Diqing cattle based on mtDNA data (Nie et al., 1999; Yu et al., 1999). In this study, both Diqing cattle and Zhongdian yak were collected from Shangri-La, Diqing Prefecture. The bidirectional genetic introgression between Diqing cattle and Zhongdian yak was detected, and this result was similar to previously reported results based on the genetic introgression between Tibetan cattle and yak (Chen et al., 2018; Wu et al., 2018). Simultaneously, the enrichment analysis showed that the introgressed genes from the Zhongdian yak to Diqing cattle were mainly enriched in immune-related pathways, being different from those of previous studies (Chen et al., 2018). Their studies also detected some pathways related to diseases, speculating that the genes related to the disease were not subject to strong selection. Three introgressed genes (*ENPEP*, *FLT1*, and *PIK3CA*) related to high-altitude and hypoxia adaptations were detected in Diqing cattle, of which the *ENPEP* gene has been reported in high-altitude adaptability studies of the Andean (Eichstaedt et al., 2015) and Tibetan goats (Wang et al., 2016) and Tibetan sheep (Wei et al., 2016). The previously reported common *EGLN1* gene (Chen et al., 2018; Wu et al., 2018) was not detected in our study. Here, the *PIK3CA* gene was an important component of the hypoxia-inducible factor 1 (HIF-1) pathway (Shimomura et al., 2013). These genes detected may have contributed to the adaptation of Diqing cattle to the high-altitude and hypoxia environment.

The introgression event was also found between the Zhongdian yak and Tibetan cattle in our study, being similar to the previous study on genetic components from the yak introgressed into Tibetan cattle (Chen et al., 2018; Wu et al., 2018). The 30 common genes under positive selection were detected in Tibetan cattle, which were enriched in the chemokine signaling pathway, indicating that immune-related pathways probably play an important role in resisting diseases and maintaining energy balance in high-altitude environments. Interestingly, the *EPAS1* gene was detected by using the XP-EHH method, which was congruent with the previous study using *F*_ST_, XP-EHH, and ΔDAF methods based on re-sequencing technology (Wu et al., 2019). Studies showed that the *EPAS1* gene was under positive selection and had undergone convergent evolution in Tibetan dogs, Tibetans, and Tibetan pigs (Li et al., 2014; Wang et al., 2014; Wu et al., 2019). Additionally, the *CDC42* and *SLC39A2* genes were also found in Tibetan cattle. Among *CDC42* related to the morphology, adhesion, migration, and erythropoiesis of hematopoietic stem cells (Wang et al., 2006; Yang et al., 2007), this gene can mediate bmp-induced sprouting angiogenesis (Wakayama et al., 2015). *CDC42* was under positive selection and enriched with vascular endothelial growth factor (VEGF) for Tibetan goats (Jin et al., 2020). *SLC39A2* and *SLC9A6* genes were related to heart functions (Du et al., 2019), which have also been reported in Yunnan golden monkeys (Zhou et al., 2016). It is speculated that the regulation of genes related to hypoxia response, vascular development and erythropoiesis, and heart functions may have extremely important significance for the high-altitude adaptation of Tibetan cattle by the aforementioned comparative studies.

In conclusion, we used the high-density BovineHD genotyping arrays to assess the population structure and genetic introgression of *Bos* species. The genetic relationships between the zebu, yak, and gaur mirrored their geographical origins. Furthermore, it confirmed that there were at least three ancestral components of the European taurine, East Asian taurine, and Indian indicine in domestic cattle. We found the bidirectional genetic introgression between Diqing cattle and Zhongdian yak. Especially, the Zhongdian yak introgression into Diqing cattle will help the latter to quickly adapt to the high-altitude and hypoxia environment. Furthermore, we also found that the genetic components of the Zhongdian yak had introgressed into Tibetan cattle and detected some genes (*CDC42*, *SLC39A2*, and *EPAS1*) associated with high-altitude adaptation by using three methods of selection signatures (*F*_ST_, XP-EHH, and XP-CLR). The functions of these genes might have great significance for high-altitude adaptation of Tibetan cattle. These results showed that genetic introgression was one of the important ways for the environmental adaptation of domestic cattle. Future studies are needed to validate these results by using whole-genome re-sequencing and functional verification studies.

## Data Availability

The data sets presented in this study can be found in online repositories. The names of the repository/repositories and accession number(s) can be found at: https://datadryad.org/stash/dataset/doi:10.5061/dryad.h9w0vt4k8.
