# Supplementary material for: Whole-Genome Analysis Deciphers Population Structure and Genetic Introgression Among Bovine Species
Source: Front Genet. 2022 May 27;13:847492. doi: 10.3389/fgene.2022.847492 (PMC9197319; doi:10.3389/fgene.2022.847492)
Supplement: Supplementary file 1 [file DataSheet1.docx]

***Supplementary material***

**Table S1** The *f*3 statistics of Diqing cattle and other bovine species.

| Source1 | Source2 | Target | *f*3 statistic | Standard error | Z-score |
| --- | --- | --- | --- | --- | --- |
| **Zhongdian yak** | **Holstein** | **Diqing cattle** | **-0.005407** | **0.000940** | **-5.754** |
| Tibetan yak | Holstein | Diqing cattle | -0.005079 | 0.000981 | -5.177 |
| Maiwa yak | Holstein | Diqing cattle | -0.004998 | 0.000967 | -5.171 |
| Tibetan cattle | Holstein | Diqing cattle | 0.016027 | 0.000459 | 34.891 |
| Myanmar cattle | Holstein | Diqing cattle | 0.004763 | 0.000753 | 6.328 |
| Indian zebu | Holstein | Diqing cattle | 0.006322 | 0.000751 | 8.418 |
| Gaur | Holstein | Diqing cattle | 0.000542 | 0.000944 | 0.574 |

**Table S2** The *D* statistic of Diqing cattle and other three bovine species. A negative *D* value shows the gene flow from Y to X, and a positive *D* value shows the gene flow from Y to W.

| W | X | Y | Z | *D* statistic | Z-score |
| --- | --- | --- | --- | --- | --- |
| **Holstein** | **Diqing cattle** | **Zhongdian yak** | **Gaur** | **-0.047** | **-13.164** |
| Holstein | Diqing cattle | Tibetan yak | Gaur | -0.048 | -12.284 |
| Holstein | Diqing cattle | Maiwa yak | Gaur | -0.047 | -11.609 |
| Holstein | Diqing cattle | Tibetan cattle | Gaur | 0.043 | 17.142 |
| Holstein | Diqing cattle | Myanmar cattle | Gaur | 0.017 | 7.406 |
| Holstein | Diqing cattle | Bangladesh zebu | Gaur | 0.021 | 9.145 |
| Holstein | Diqing cattle | Indian zebu | Gaur | 0.023 | 9.939 |

**Table** **S3** The *f*3 statistics of Zhongdian yak and other bovine species.

| Source1 | Source2 | Target | *f*3 statistic | Standard error | Z-score |
| --- | --- | --- | --- | --- | --- |
| **Diqing cattle** | **Tibetan yak** | **Zhongdian yak** | **-0.046459** | **0.002583** | **-17.987** |
| Tibetan cattle | Tibetan yak | Zhongdian yak | -0.045799 | 0.002592 | -17.669 |
| Holstein | Tibetan yak | Zhongdian yak | -0.044309 | 0.002767 | -16.011 |
| Indian zebu | Tibetan yak | Zhongdian yak | -0.023103 | 0.001824 | -12.663 |
| Myanmar cattle | Tibetan yak | Zhongdian yak | -0.022601 | 0.001815 | -12.451 |
| Bangladesh zebu | Tibetan yak | Zhongdian yak | -0.022021 | 0.001865 | -11.808 |
| Gaur | Tibetan yak | Zhongdian yak | -0.001965 | 0.001498 | -1.312 |
| **Diqing cattle** | **Maiwa yak** | **Zhongdian yak** | **-0.042314** | **0.002999** | **-14.108** |
| Tibetan cattle | Maiwa yak | Zhongdian yak | -0.041552 | 0.003122 | -13.309 |
| Holstein | Maiwa yak | Zhongdian yak | -0.039625 | 0.003456 | -11.465 |
| Indian zebu | Maiwa yak | Zhongdian yak | -0.022077 | 0.002335 | -9.454 |
| Myanmar cattle | Maiwa yak | Zhongdian yak | -0.022111 | 0.002234 | -9.898 |
| Bangladesh zebu | Maiwa yak | Zhongdian yak | -0.020703 | 0.002359 | -8.776 |
| Gaur | Maiwa yak | Zhongdian yak | -0.003411 | 0.001593 | -2.141 |

**Table S4** The D statistic of Zhongdian yak and other three bovine species. A negative D value shows the gene flow from Y to X, and a positive D value shows the gene flow from Y to W.

| W | X | Y | Z | *D* statistic | Z-score |
| --- | --- | --- | --- | --- | --- |
| **Tibetan yak** | **Zhongdian yak** | **Diqing cattle** | **Gaur** | **-0.134** | **-15.981** |
| Tibetan yak | Zhongdian yak | Tibetan cattle | Gaur | -0.131 | -15.715 |
| Tibetan yak | Zhongdian yak | Holstein | Gaur | -0.122 | -14.505 |
| Tibetan yak | Zhongdian yak | Indian zebu | Gaur | -0.077 | -12.094 |
| Tibetan yak | Zhongdian yak | Myanmar cattle | Gaur | -0.076 | -12.102 |
| Tibetan yak | Zhongdian yak | Bangladesh zebu | Gaur | -0.073 | -11.358 |
| Tibetan yak | Zhongdian yak | Maiwa yak | Gaur | 0.0554 | 9.526 |

**Table S5** The fragments number, fragments length and proportion of Zhongdian yak introgressed into Diqing cattle.

|  |  |  | Introgression fragments length | | | |  |  |
| --- | --- | --- | --- | --- | --- | --- | --- | --- |
| Samples | Count |  | minimum | maximum | average | Sum |  | Proportion |
| DQ01 | 71 |  | 28,622 | 2,166,738 | 897,746 | 63,739,970 |  | 1.34% |
| DQ02 | 367 |  | 28,622 | 2,814,305 | 996,293 | 365,639,815 |  | 7.68% |
| DQ03 | 81 |  | 28,622 | 1,909,381 | 808,507 | 65,489,080 |  | 1.38% |
| DQ04 | 182 |  | 28,622 | 3,436,672 | 947,563 | 172,456,583 |  | 3.62% |
| DQ05 | 245 |  | 217,063 | 2,814,305 | 1,012,511 | 248,065,308 |  | 5.21% |
| DQ06 | 186 |  | 28,622 | 3,697,256 | 863,585 | 160,626,824 |  | 3.37% |
| DQ07 | 56 |  | 28,622 | 2,281,721 | 950,912 | 53,251,108 |  | 1.12% |
| DQ08 | 232 |  | 198,428 | 2,662,441 | 957,910 | 222,235,235 |  | 4.67% |
| DQ09 | 50 |  | 28,622 | 2,961,245 | 1,019,213 | 50,960,678 |  | 1.07% |
| DQ10 | 93 |  | 89,556 | 2,166,649 | 799,829 | 74,384,128 |  | 1.56% |
| DQ11 | 61 |  | 217,063 | 1,931,427 | 853,358 | 52,054,883 |  | 1.09% |
| DQ12 | 70 |  | 28,622 | 2,814,305 | 831,657 | 58,216,058 |  | 1.22% |
| DQ13 | 64 |  | 28,622 | 2,281,721 | 662,018 | 42,369,189 |  | 0.89% |
| DQ14 | 84 |  | 28,622 | 2,246,117 | 790,196 | 66,376,504 |  | 1.39% |
| DQ15 | 76 |  | 28,622 | 2,961,245 | 904,646 | 68,753,153 |  | 1.44% |
| DQ16 | 47 |  | 28,622 | 1,768,898 | 850,818 | 39,988,468 |  | 0.84% |

**Table S6** The fragments number, fragments length and proportion of Diqing cattle introgressed into Zhongdian yak.

|  |  |  | Introgression fragments length | | | |  |  |
| --- | --- | --- | --- | --- | --- | --- | --- | --- |
| Samples | Count |  | Minimum | Maximum | Average | Sum |  | Proportion |
| ZDY01 | 363 |  | 226,137 | 2,441,640 | 1,029,489 | 373,704,654 |  | 7.84% |
| ZDY02 | 321 |  | 86,475 | 3,791,115 | 951,726 | 305,504,273 |  | 6.41% |
| ZDY03 | 572 |  | 25,220 | 2,662,258 | 993,639 | 568,361,672 |  | 11.92% |
| ZDY04 | 341 |  | 86,475 | 2,542,246 | 960,116 | 327,399,597 |  | 6.87% |
| ZDY05 | 1012 |  | 24,729 | 3,791,115 | 942,319 | 953,626,859 |  | 21.25% |
| ZDY06 | 69 |  | 86,475 | 2,191,656 | 789,806 | 54,496,681 |  | 1.14% |
| ZDY07 | 66 |  | 475,237 | 1,659,719 | 949,518 | 62,668,236 |  | 1.31% |
| ZDY08 | 61 |  | 475,237 | 2,326,626 | 1,020,630 | 62,258,458 |  | 1.31% |
| ZDY09 | 63 |  | 199,859 | 1,463,025 | 747,484 | 47,091,541 |  | 0.99% |
| ZDY10 | 52 |  | 445,120 | 1,767,783 | 902,823 | 46,946,832 |  | 0.98% |
| ZDY11 | 158 |  | 86,475 | 2,834,269 | 997,502 | 157,605,434 |  | 3.31% |
| ZDY12 | 37 |  | 471,788 | 2,367,371 | 1,070,907 | 39,623,584 |  | 0.83% |
| ZDY13 | 72 |  | 199,859 | 1,896,728 | 902,112 | 64,952,093 |  | 1.36% |

**Table** **S7** The GO and KEGG enrichment analyses of introgression genes of Zhongdian yak introgression into Diqing cattle.

| Category | Term | Count | PValue |
| --- | --- | --- | --- |
| MF | GO:0048020~CCR chemokine receptor binding | 13 | 1.07E-06 |
| MF | GO:0008270~zinc ion binding | 143 | 3.94E-04 |
| MF | GO:0008009~chemokine activity | 13 | 4.44E-04 |
| MF | GO:0019003~GDP binding | 13 | 2.55E-03 |
| MF | GO:0004672~protein kinase activity | 28 | 3.69E-03 |
| MF | GO:0004519~endonuclease activity | 9 | 5.29E-03 |
| BP | GO:0002548~monocyte chemotaxis | 15 | 1.44E-06 |
| BP | GO:0048247~lymphocyte chemotaxis | 13 | 1.56E-06 |
| BP | GO:0071347~cellular response to interleukin-1 | 18 | 1.41E-05 |
| BP | GO:0071346~cellular response to interferon-gamma | 15 | 3.14E-05 |
| BP | GO:0071356~cellular response to tumor necrosis factor | 18 | 2.84E-04 |
| BP | GO:0006954~inflammatory response | 44 | 3.89E-04 |
| BP | GO:0030593~neutrophil chemotaxis | 15 | 8.02E-04 |
| BP | GO:0090501~RNA phosphodiester bond hydrolysis | 5 | 8.98E-04 |
| BP | GO:0070374~positive regulation of ERK1 and ERK2 cascade | 29 | 1.20E-03 |
| BP | GO:0070098~chemokine-mediated signaling pathway | 14 | 1.33E-03 |
| BP | GO:0043922~negative regulation by host of viral transcription | 7 | 2.27E-03 |
| BP | GO:0033674~positive regulation of kinase activity | 6 | 3.54E-03 |
| BP | GO:0090201~negative regulation of release of cytochrome c from mitochondria | 7 | 3.58E-03 |
| BP | GO:0007030~Golgi organization | 16 | 3.66E-03 |
| BP | GO:0060322~head development | 5 | 5.16E-03 |
| BP | GO:0060763~mammary duct terminal end bud growth | 4 | 6.10E-03 |
| BP | GO:0016050~vesicle organization | 8 | 6.28E-03 |
| BP | GO:0010212~response to ionizing radiation | 11 | 9.26E-03 |
| CC | GO:0005773~vacuole | 8 | 1.56E-04 |
| CC | GO:0005768~endosome | 31 | 1.08E-03 |
| CC | GO:0016023~cytoplasmic, membrane-bounded vesicle | 19 | 7.15E-03 |
| CC | GO:0005925~focal adhesion | 54 | 7.17E-03 |
| CC | GO:0005623~cell | 16 | 7.24E-03 |
| KEGG | bta04062:Chemokine signaling pathway | 35 | 4.82E-04 |
| KEGG | bta04670:Leukocyte transendothelial migration | 23 | 3.06E-03 |
| KEGG | bta04360:Axon guidance | 23 | 7.70E-03 |
| KEGG | bta04071:Sphingolipid signaling pathway | 22 | 9.70E-03 |

**Table S8** The *f*3 statistics of Tibetan cattle and other bovine species.

| Source1 | Source2 | Target | *f*3 statistic | Standard error | Z-score |
| --- | --- | --- | --- | --- | --- |
| **Zhongdian yak** | **Holstein** | **Tibetan cattle** | **-0.005210** | **0.000758** | **-6.877** |
| Tibetan yak | Holstein | Tibetan cattle | -0.005000 | 0.000788 | -6.343 |
| Maiwa yak | Holstein | Tibetan cattle | -0.004930 | 0.000784 | -6.291 |
| Gaur | Holstein | Tibetan cattle | -0.001380 | 0.000722 | -1.907 |
| Diqing cattle | Holstein | Tibetan cattle | 0.000317 | 0.000337 | 0.941 |
| Myanmar cattle | Holstein | Tibetan cattle | 0.000767 | 0.000574 | 1.335 |
| Indian zebu | Holstein | Tibetan cattle | 0.000468 | 0.000571 | 0.820 |

**Table S9** The *D* statistics of Tibetan cattle and other bovine species. A negative D value shows the gene flow from Y to X, and a positive D value shows the gene flow from Y to W.

| W | X | Y | Z | *D* statistic | Z-value |
| --- | --- | --- | --- | --- | --- |
| **Holstein** | **Tibetan cattle** | **Zhongdian yak** | **Gaur** | **-0.0321** | **-10.250** |
| Holstein | Tibetan cattle | Tibetan yak | Gaur | -0.0327 | -9.435 |
| Holstein | Tibetan cattle | Maiwa yak | Gaur | -0.0318 | -8.850 |
| Holstein | Tibetan cattle | Diqing cattle | Gaur | 0.0051 | 2.438 |
| Holstein | Tibetan cattle | Indian zebu | Gaur | 0.0077 | 3.794 |
| Holstein | Tibetan cattle | Bangladesh zebu | Gaur | 0.0064 | 3.185 |
| Holstein | Tibetan cattle | Myanmar cattle | Gaur | 0.0091 | 4.558 |

**
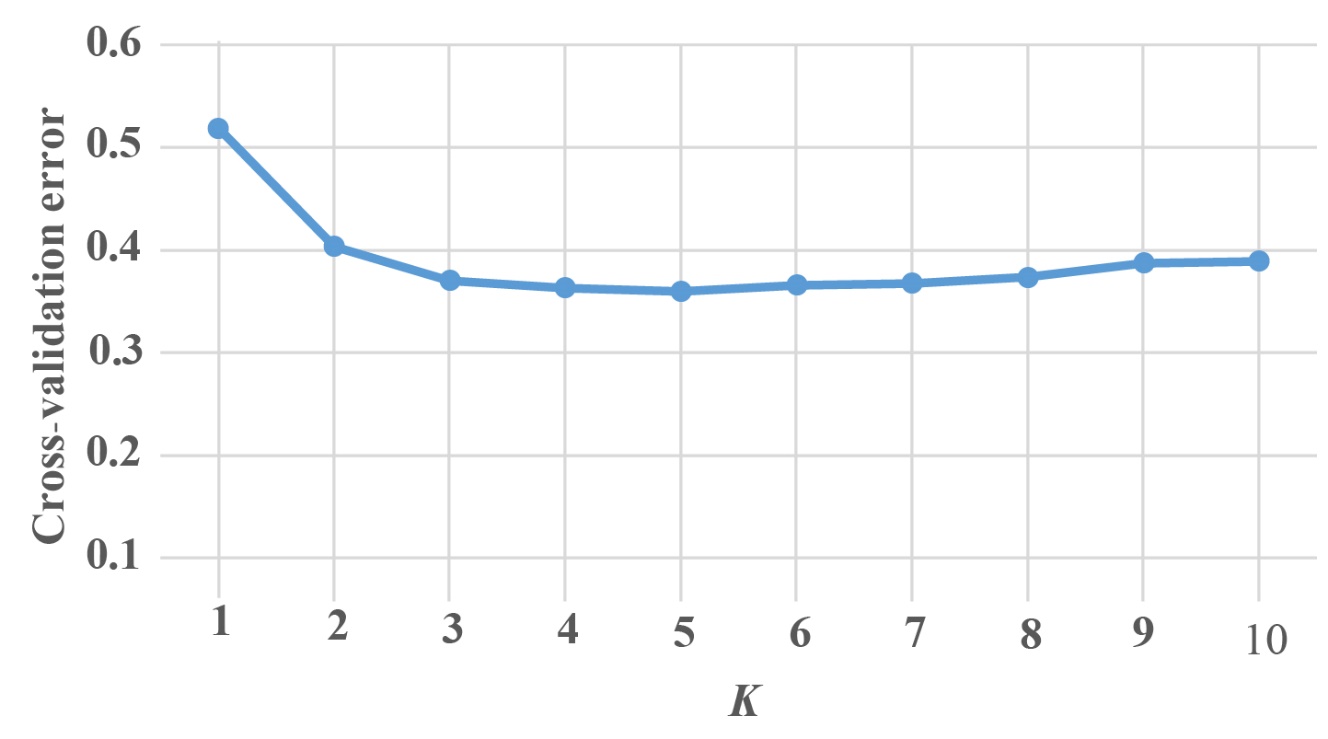
**

**Figure S1.** Cross-validation error calculated for each value of K=1–10 in ADMIXTURE program.

**
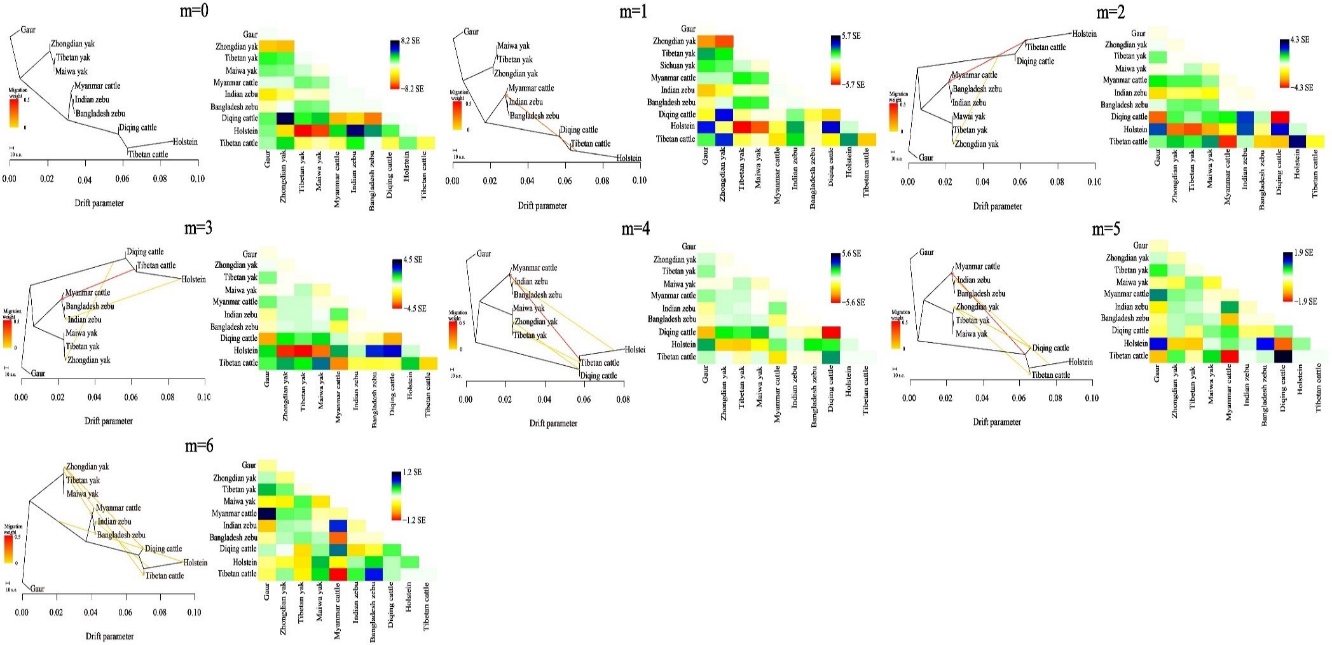
**

**Figure S2.** Genetic migration inferred by TreeMix among ten cattle population (migration number from 1~ 6, 0 means no migration).
